# Aluminum Nanoholes for Optical Biosensing

**DOI:** 10.3390/bios5030417

**Published:** 2015-07-09

**Authors:** Carlos Angulo Barrios, Víctor Canalejas-Tejero, Sonia Herranz, Javier Urraca, María Cruz Moreno-Bondi, Miquel Avella-Oliver, Ángel Maquieira, Rosa Puchades

**Affiliations:** 1 Instituto de Sistemas Optoelectrónicos y Microtecnología (ISOM), ETSI Telecomunicación, Universidad Politécnica de Madrid, Ciudad Universitaria s/n, Madrid 28040, Spain; E-Mail: victorcanalejas@isom.upm.es; 2 Departamento de Tecnología Fotónica y Bioingeniería (TFB), ETSI Telecomunicación, Universidad Politécnica de Madrid, Ciudad Universitaria s/n, Madrid 28040, Spain; 3 Departamento de Química Analítica, Universidad Complutense de Madrid, Ciudad Universitaria s/n, Madrid 28040, Spain; E-Mails: sherranz@quim.ucm.es (S.H.); jurracar@quim.ucm.es (J.U.); mcmbondi@ucm.es (M.C.M.-B.); 4 CEI Campus Moncloa, UCM-UPM, Avda. Complutense s/n, Madrid 28040, Spain; 5 Instituto de Reconocimiento Molecular y Desarrollo Tecnológico (IDM), Departamento de Química, Universidad Politécnica de Valencia, Camino de Vera s/n, Valencia 46022, Spain; E-Mails: miavol@upvnet.upv.es (M.A.-O.); amaquieira@qim.upv.es (A.M.); rpuchades@qim.upv.es (R.P.)

**Keywords:** aluminum, metal nanoholes, nanohole arrays, surface plasmon resonance, optical biosensing, nanopatterning, transfer printing, molecularly imprinted polymer, photopolymerization

## Abstract

Sub-wavelength diameter holes in thin metal layers can exhibit remarkable optical features that make them highly suitable for (bio)sensing applications. Either as efficient light scattering centers for surface plasmon excitation or metal-clad optical waveguides, they are able to form strongly localized optical fields that can effectively interact with biomolecules and/or nanoparticles on the nanoscale. As the metal of choice, aluminum exhibits good optical and electrical properties, is easy to manufacture and process and, unlike gold and silver, its low cost makes it very promising for commercial applications. However, aluminum has been scarcely used for biosensing purposes due to corrosion and pitting issues. In this short review, we show our recent achievements on aluminum nanohole platforms for (bio)sensing. These include a method to circumvent aluminum degradation—which has been successfully applied to the demonstration of aluminum nanohole array (NHA) immunosensors based on both, glass and polycarbonate compact discs supports—the use of aluminum nanoholes operating as optical waveguides for synthesizing submicron-sized molecularly imprinted polymers by local photopolymerization, and a technique for fabricating transferable aluminum NHAs onto flexible pressure-sensitive adhesive tapes, which could facilitate the development of a wearable technology based on aluminum NHAs.

## 1. Introduction

Nanophotonic devices are nanostructures that facilitate the generation, propagation, manipulation, and detection of light [1]. They can be employed to create highly localized optical fields on the nanoscale, allowing strong enhancement of light-matter interaction. Because of this, one of the most promising applications of these structures is their use as very sensitive transducers for optical biosensing. Additional advantages of nanophotonic devices for this purpose are: Small footprint—which permits large scale integration for multiplex analysis based on sensor arrays—and possibility of mass production by mature micro- and nano-processing techniques, as those employed in the microelectronics industry. In this respect, two major micro- and nano-photonic platforms have excelled: Si-based integrated photonic devices [2] and plasmonic nanostructures based on metal sub-wavelength features [3]. Remarkable examples of the former are Mach-Zehnder [4] and Young interferometers [5], microring [6], Fabry-Perot [7], and photonic crystal [8] resonators, whereas metal nanoparticles [9] and nanoholes in metal films [10] are the most representative plasmonic nanostructures applied to the field of biosensors.

Concerning nanoplasmonic devices, those based on metallic subwavelength holes are particularly appealing because of their geometrical simplicity and the possibility of being reliably and uniformly mass-produced through lithographic means [11,12], facilitating massive parallelism. These plasmonic transducers can be typically configured as either nanohole arrays (NHAs) that function as metal gratings capable of exciting surface plasmon polaritons (SPPs) Bloch waves [13] or optically isolated nanoholes. Numerous demonstrations of NHA-based chemical and biochemical sensors can be found in the literature [14]. The principle of operation of these devices relies on the sensitivity of SPP resonances to the dielectric materials in contact with the metal surface, thus refractive index changes can be optically measured as spectral feature shifts or intensity variations. NHAs are ideal structures for the implementation of multiplexed-detection platforms with integrated microfluidics because of their high spatial density and smaller sensing areas than those used in conventional surface plasmon resonance (SPR) coupling techniques like those based on prisms. Regarding isolated nanoholes, these can also excite surface plasmons [13] and act as metal-clad optical waveguides [12,15]. An outstanding biosensing manifestation of the latter was the achievement of single molecule detection by fluorescence correlation spectroscopy at biologically relevant concentrations [15].

The majority of demonstrated plasmonic metal NHAs have been fabricated in Au and Ag films. This is because these materials have low optical losses in the visible and near-infrared ranges, in addition to a high chemical stability. The latter property is particularly important for biochemical uses and mainly applicable to Au; Ag has a poorer environmental stability and usually requires a protective overlayer of a dielectric material, such as silica [16] or alumina [17]. The excellent plasmonic properties of these metals are however counteracted by their high cost, which limits large scale commercialization. A very attractive alternative to noble metals is Al, which is approximately 25,000 and 425 times cheaper than Au and Ag, respectively, easy to manufacture and process and has material properties that enable plasmon resonances in a broad optical band [18,19]. However, Al has been scarcely taken into account for the implementation of SPR biosensors mainly because of challenges from oxidation and material degradation (corrosion and pitting), which are particularly critical when thin Al films are concerned.

In this paper, we review our recent research results on Al nanoholes for (bio)sensing applications. In Section 2 a passivation process for Al NHAs fabricated on glass substrates and its effectiveness for biosensing in aqueous media are described. Section 3 depicts a refinement of the technology developed for glass substrates to be compatible with polycarbonate (PC) supports from compact discs (CDs), and the demonstration of the suitability of the resulting Al NHA on CD-based label-free optical biosensing platform. Section 4 presents a direct and easy method to transfer Al NHAs from PC surfaces onto pressure-sensitive adhesive (Scotch) tapes, allowing the fabrication of Al NHA-based sensors on flexible supports. Section 5 deals with the use of isolated Al nanoholes as optical waveguides for synthesizing molecularly imprinted polymers (MIPs) with submicron lateral dimensions by localized photopolymerization. Finally, conclusions are collected in Section 6.

## 2. Passivation of Al NHAs

**Figure 1:**
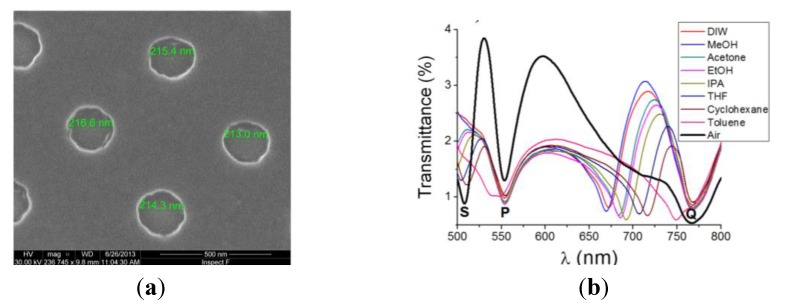
(**a**) Scanning electron microscope (SEM) photograph of a fabricated 500-nm-period Al NHA. Good surface and hole diameter uniformity is observed; (**b**) Measured spectral transmittances of a 500-nm-period Al NHA on glass immersed in different fluids. S-wavelength is related to the SPP resonance at the interface between the superstrate (air, liquids) and the metal: Its position redshifts from ~507 nm (air) to ~(670–750) nm (liquids) as the superstrate refractive index increases (bulk refractive index sensitivity = 487 nm/RIU). P- and Q-wavelengths are related to metal/substrate SPP resonances.

We employed the following process flow to fabricate Al NHAs on glass substrates: (1) deposition of a 100-nm-thick Al layer on glass supports by electron-beam thermal evaporation; (2) ZEP520 resist coating of the Al surface, followed by electron-beam lithography (EBL) exposure to define the nanoholes and subsequent resist development; (3) inductively coupled plasma (ICP) etching of the Al film using the patterned EBL resist as a mask; and (4) rinsing of the etched Al surface to remove residual chloride ions. Detailed description of the fabrication process can be found in [20]. Figure 1 shows representative results concerning the surface morphology and optical characterization of a 500-nm-period Al NHA. The measured transmittance spectrum for unpolarized, normally incident light exhibit three main resonance features, referred to as their respective minima: S-wavelength (~507 nm), P-wavelength (~550 nm) and Q-wavelength (~770 nm). These values were in good agreement with both, finite difference time domain (FDTD) simulations and the predicted SPP resonances by the well-known SPP grating-coupling equation:

(1) $$\lambda_{SPP}\approx \frac{a}{\sqrt{i^{2}+j^{2}}}\sqrt{\frac{\varepsilon_{d}\varepsilon_{m}}{\varepsilon_{d}+\varepsilon_{m}}}$$

where λ_SPP_ is the wavelength of the SPP resonance, *a* is the array period, *i* and *j* are integers and ε*_m_* and ε_d_ are the dielectric functions of the metal and dielectric medium, respectively. According to this equation, P- and Q-wavelengths correspond to SPP resonances at the glass/metal interface for *i* = ±1, *j* = ±1 and *i* = ±1, *j* = 0 (or *vice versa*), respectively. The S-wavelength (λ_S_) is related to the *i* = ±1, *j* = 0 (or *vice versa*) SPP resonance at the air/metal interface; therefore, both, bulk (optical changes in the whole SPP evanescent field region) and surface (optical changes in the close proximity to the NHA surface) sensing can be performed by measuring λ_S_.

To prepare the nanopatterned films for biosensing tests in aqueous solutions, the issue of Al oxidation was dealt with a passivation process consisting of exposing the devices to an oxygen plasma [20]. The purpose of this treatment was to produce an oxide protecting layer more resistant chemically—particularly, against oxidizing agents—than the usually present native oxide. By monitoring the optical response (variation of λ_S_) of the plasma-exposed Al NHAs as a function of the plasma treatment time and the immersion time in deionized water (DIW) (pH = 5.7), it was found that a plasma treatment of ~40 min led to an oxidized surface that provided protection against further oxidation and, simultaneously, did not increase the oxide film thickness significantly. A too-thick oxide layer would decrease the surface sensitivity of the NHA since the maximum sensitivity is achieved at the metal/dielectric media interface. Although the passivation process does not completely avoid oxidation in aqueous media, it reduces the oxidation rate to a value low enough (~0.08 nm/h) to allow biosensing tests to be reliably achieved in a reasonable time. The experimental bulk refractive index sensitivity of an oxygen plasma treated Al NHA (Figure 1b) was 487 nm/RIU (RIU ≡ refractive index unit), which compares well to those of similar NHAs made in Au [21] and Ag [22] films.

As a proof-of-concept, we employed the passivated Al NHAs on glass substrates as optical transducers for performing a label-free immunoassay for biotin analysis. The measuring principle was based on a competitive inhibition assay between the biotin-dextran-lipase conjugates immobilized onto the Al NHA surface and biotin present in a sample solution for a limited number of antibiotin antibody binding sites. The devices were responsive to receptor immobilization, and gave a higher signal (λ_S_ shift) in the absence than in the presence of the analyte, demonstrating good biosensing performance. Despite the several incubations in aqueous solutions involved in these experiments (some of them as long as 60 min), no evidence of aluminum degradation was observed. This supports the effectiveness of our passivation process, which provides protection against Al oxidation and corrosion and generates a surface capable to be derivatized by well-established silane chemistry for bioreceptor immobilization.

## 3. Al NHAs on Polycarbonate Compact Discs

We applied the technology described in the previous section to the fabrication of Al NHAs on CD substrates made of PC [23]. The use of CD supports along with CD players has been shown to be a suitable and cost-effective biosensing optical platform for performing microarray assays [24]. Since NHAs are ideal structures for array configurations, their implementation on CD surfaces appears to be a promising approach to develop label-free high-density microarray analysis automation by portable read-out equipment.

Using PC, instead of glass, as the substrate material implies considering fabrication precautions in order not to degrade the substrate during device processing. In particular, the critical PC properties that must be taken into account are its glass transition temperature (~147 °C) and its reactivity with solvents, such as EBL resist developers. The technology was therefore adapted by introducing three key processes: (i) EBL resist baking at 120 °C for 10 min (instead of the typical bake conditions for ZEP520 resist: 180 °C for 2 min) to allow the resist solvent to evaporate while keeping the PC temperature below its glass transition temperature; (ii) adhesion of a removable protective plastic film onto the PC substrate backside to prevent that surface from reacting with the EBL developer; and (iii) the O_2_ plasma-based Al surface passivation process was carried out in several separate and consecutive short steps to avoid substrate overheating. By adopting these PC-compatible processes, the substrate transparency and integrity were preserved throughout the whole fabrication process.

**Figure 2:**
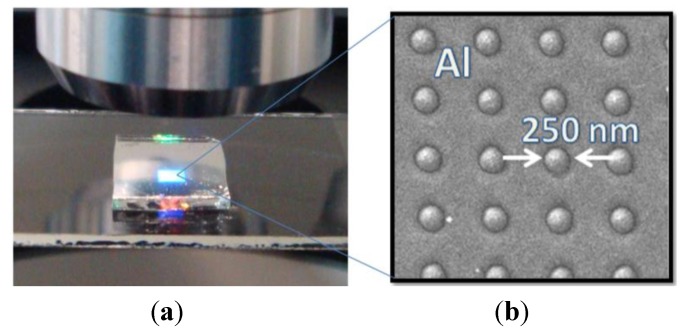
(**a**) A 500-nm-period Al NHA on a 1.2-mm-thick PC substrate illuminated through a microscope objective. Light diffraction is clearly observed; (**b**) SEM photograph of the nanopatterned Al surface revealing good hole diameter uniformity.

**Figure 3:**
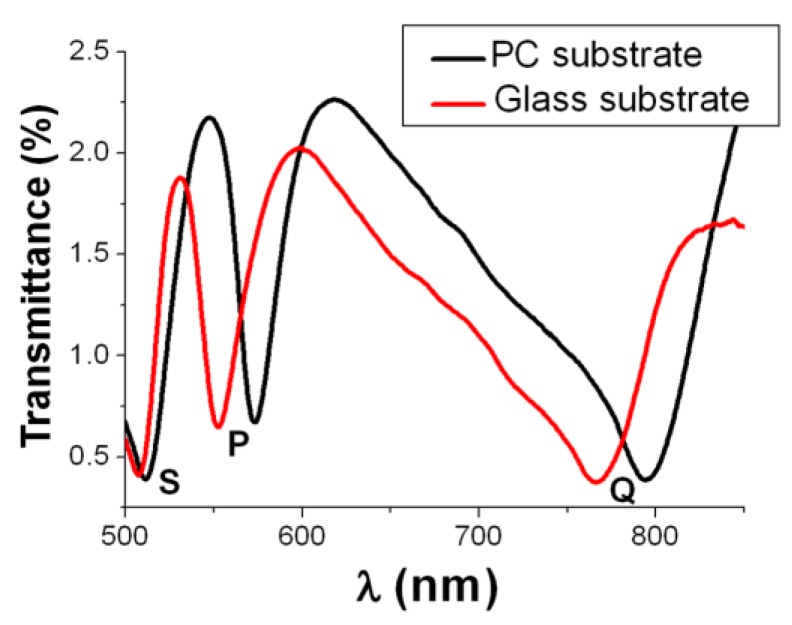
Experimental spectral transmittance of 500-nm-period Al NHAs on polycarbonate and glass substrates. The spectra exhibit three similar transmission minima: S-wavelength is associated to the superstrate (air)/metal interface SPP resonance, whereas P- and Q-wavelengths are related to substrate/metal SPP resonances.

The fabricated 500-nm-period Al NHAs exhibited both, good surface and hole diameter (~250 nm) uniformity and clear optical diffraction properties (Figure 2). The shape of the measured spectral transmittance was similar to that of NHAs fabricated on glass substrates (Figure 3). Three transmission minima appear due to SPPs at air/metal (S-wavelength) and substrate/metal interfaces (P- and Q-wavelengths). Substrate/metal SPPs occur at different wavelengths than those measured for the glass substrate because of the differences in refractive indexes between PC (~1.58) and glass (~1.52). Following the same experimental protocol as that for the NHAs on glass, the viability of the Al NHA-CD platform for label-free optical biosensing in aqueous media was also demonstrated [23].

**Figure 4:**
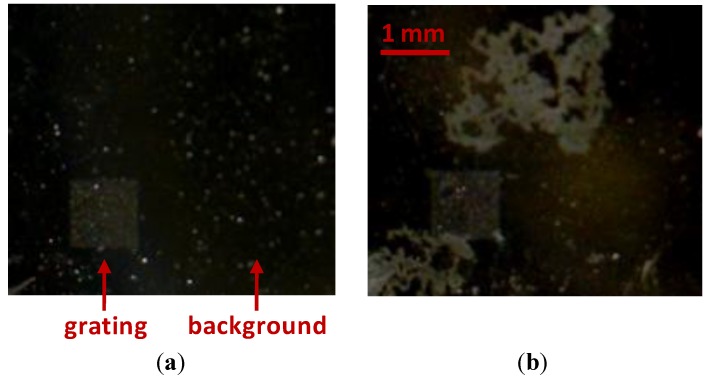
Example of oxidized Al surfaces degradation, before (**a**) and after (**b**) interacting with PBS buffer.

In addition to the aforementioned immunoassay, we studied the sensitivity of the oxidized Al surfaces to chemical parameters, such as pH and concentration of certain ions and tensioactives. This study served as a preliminary assessment for determining the feasibility of different buffers for Al NHAs biosensing. For that, the degradation of the material was macroscopically evaluated after placing different aqueous solutions on Al NHAs (Figure 4). Considering the results shown in Table 1, HEPES and HEPES-T were selected to address biorecognition assays on Al NHAs.

**Table 1:** Qualitative assessment of Al NHAs stability against different solutions.

Tensioactive	Deg. ^a^	Buffer	Deg. ^a^	Buffer-T ^b^	Deg. ^a^
Tween 20 (0.05%)	Yes	Tris (20 mM, pH 8.5)	No	Tris-T	No
Span 20 (0.05%)	No	PBS (10 mM, pH 7.5)	Yes	PBS-T	Yes
CTAB (0.01 M)	Yes	HEPES (25 mM, pH 7.5)	No	HEPES-T	No
SDS (0.01 M)	No	SSC (15 mM, pH 7)	Yes	SSC-T	Yes
		Citrate (25 mM, pH 5.5)	No	Citrate-T	No

^a^ Surface degradation determined macroscopically. ^b^ Buffers with Tween 20 at 0.05% (*v*/*v*).

**Figure 5:**
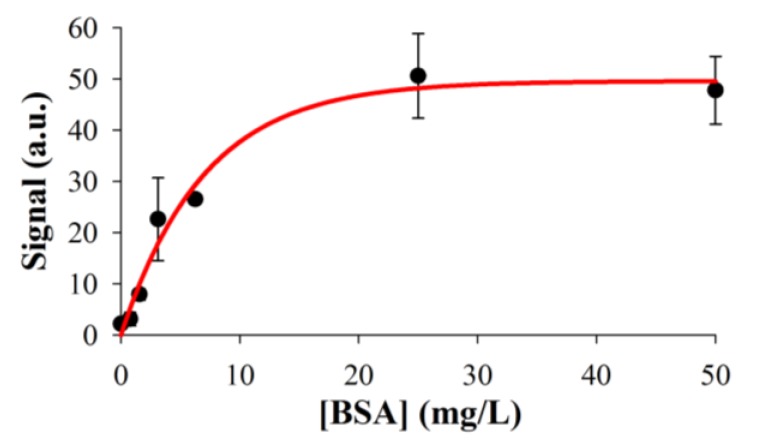
Results of the biorecognition assay model system for BSA detection on oxidized Al, measured by scanner fluorescence. Experimental data fit to an exponential rise to max curve (*R*^2^ = 0.978).

Additionally, the functionalization of the oxidized Al surfaces and their capability for performing biorecognition assays was studied by fluorescence scanner measurements. As a model system, oxidized Al was silanized with APTMS, different concentrations of BSA were immobilized by passive adsorption and the surface was blocked with gelatin. Then, anti-BSA rabbit IgG was incubated on the chip, followed by anti-rabbit goat IgG labeled with Cy5. As shown in Figure 5, the experimental results provide insights into the viability of this strategy to carry out bioassays on Al NHAs.

**Figure 6:**
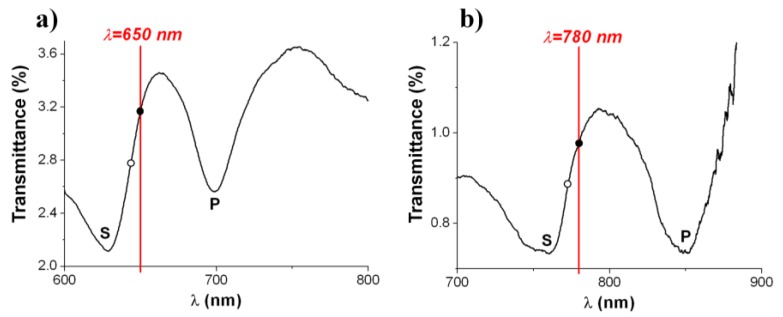
Experimental transmission spectra of 620-nm-period (**a**) and 750-nm-period; (**b**) Al NHAs on PC supports for optical intensity interrogation at 650 nm (DVD) and 780 nm (CD) wavelength, respectively. Solid black dots indicate the operating points of the transducers while hollow dots show the expected operating points of the biosensors, that is, after biological receptor immobilization on the transducers surfaces.

The demonstrated Al NHA-based biosensor on PC supports should be seen as a proof-of-concept. In order to use the laser source of a conventional CD driver for optical interrogation of NHA biosensors, optical intensity-instead of wavelength-monitoring is desirable. This entails engineering the spectral response of the NHA according to the probe interrogation wavelength to obtain an optimum performance, which can be achieved by using a proper array period. Standard driver operation wavelengths are 480 nm, 650 nm and 780 nm for Blu-ray, DVD and CD formats, respectively. Figure 6 shows the measured transmission spectra of fabricated 620-nm-period (Figure 6a) and 750-nm-period (Figure 6b) Al NHAs on PC designed to operate at 650 nm and 780 nm wavelength, respectively. Both spectra exhibit S- and P-wavelength spectral features associated to SPP resonances at air/metal and metal/substrate interfaces, respectively. The array periods have been chosen to pin the operating point (solid black dot in Figure 6) at the positive-slope side of the S-resonance. Note that the operating points are not exactly tuned at the highest slope values of the spectral curves (hollow dots in Figure 6)—where the intensity sensitivity should be maximum—but slightly redshifted. This is because the devices are aimed to have the highest sensitivity once the receptor layer has been immobilized on the sensor surface, that is, once the transducers become biosensors.

## 4. Al NHAs on Flexible Substrates

Nanopatterned thin metal films on flexible substrates is a subject of increasing interest because of the possibility of achieving plasmonic and electronic device functionalities that are not possible by using rigid substrates [25]. For example, flexible supports can be wrapped around curved and angled surfaces, enabling conformal integration of plasmonic sensors and electronics on hemispherical lenses [26] and human body parts [27]. Substrate flexibility can also lead to tunable optical devices for adaptive photonic systems [28]. This has boosted the research and development of innovative nanofabrication methods targeting simplicity, low cost, high-throughput, mass production and material compatibility.

Template stripping [29] and nanotransfer printing [30] are particularly suitable for thin metal-based device integration on flexible substrates due to their versatility and simplicity. Both techniques rely on transferring a metal nanopattern from a stamp or template to a backing layer by intimate physical contact between surfaces (stamp-metal, metal-backing). The different interface bonding strengths allow the metal nanopattern to be transferred. Patterned films of noble metals (Au, Ag) can easily be peeled from stamps due to the poor adhesion of these metals, particularly to Si-based surfaces. However, metals prone to form oxygen-rich surfaces, such as Al, exhibit higher adherence and film detachment becomes more complex, typically requiring the use of intermediate anti-adhesion layers (e.g., self-assembled monolayers (SAMs) [31] and gold films [32]).

Concerning the attachment of nanopatterned metal films onto flexible substrates, adhesive epoxy resins [29], SAM deposition [33], UV/ozone [34], controlled temperature and pressure [31] treatments are usually employed. Pressure-sensitive adhesive (PSA) tapes provide both the adhesion and the baking materials simultaneously. PSA tapes are thin, flexible, and capable to adhere to a variety of substrates by applying light pressure without the need for solvent, heat, UV or water for activation. In addition, they are low-cost and easy to use, and posses technologically relevant characteristics such as possibility of bonding dissimilar materials without incompatibility concerns, reduction of assembly times, removability, no need for surface refinishing and good uniformity and gap filling properties. Due to its versatility, PSA tapes are widely used in appliance, automotive, electronic and industrial applications, and, in recent years, nanotechnology [35,36,37].

**Figure 7:**
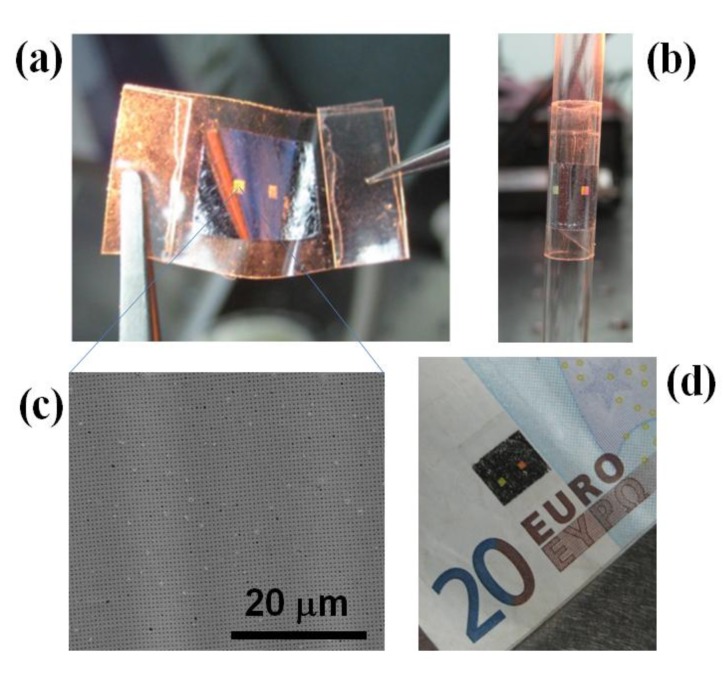
Al NHAs on Scotch tape illustrating relevant features of these devices such as flexibility, adherence and optical diffraction. (**a**) A sample picked with two tweezers; (**b**) adhered onto a glass pipette; (**c**) SEM photograph of the Al NHA surface; (**d**) sample adhered onto a note.

We have recently found that Al NHAs fabricated on PC CD surfaces according to the procedure described in Section 3 can be easily detached by using a general purpose PSA (Scotch) tape [38]. Thus, the nanopatterned Al film is directly transferred by simple tape sticking—applying finger pressure—and peeling off under ambient conditions. The transfer procedure can be watched in a video in the Supplementary Material of Reference [38]. Even though transfer parameters such as pressure, peeling angle and speed were not optimized, excellent surface uniformity and film continuity (no cracks) was attained over large areas, allowing us to observe clear optical diffractive effects as shown in Figure 7.

It should be noted that, when applied to Al NHAs on glass, the described transfer procedure did not work properly because of the high adherence of the Al films to glass surfaces. Remarkably, not even bare (non-patterned) Al films deposited on PC substrates could be acceptably detached by using the stick-and-peel method. Therefore, the successful release of the nanopatterned Al films was attributed to weakening of the metal-PC bonding strength due to particular process steps—surface cleaning before Al deposition and resist pre-bake before EBL—carried out during the NHAs fabrication on the CD substrates. Hence, no specific anti-adhesion layer was needed, simplifying the Al NHA fabrication process. The developed technology provides an Al-PC interface bonding that is both, strong enough to permit Al NHAs processing by a top-down approach (EBL and ICP) and weak enough to allow the Al NHAs to be easily peeled off by a simple transfer method with Scotch tape.

A 500-nm-period Al NHA on Scotch tape was tested as a plasmonic refractometric sensor by immersing it into different aqueous solutions of citric acid. A bulk sensitivity of 477 nm/RIU was measured, which is similar to that achieved for Al NHAs on glass (Section 2). This supports the high quality of the Al NHA transfer printing and the capability of these devices to be used as refractometric transducers for biosensing. The stickiness and flexibility of PSA tapes makes them particularly suitable for implementing ready-to-use wearable (bio)sensors capable to be applied onto a diversity of material surfaces and shapes.

## 5. Al Nanoholes for nanoMIP Synthesis

MIPs are tailor-made materials capable to exhibit selective recognition to a template molecule present during the polymerization process [39]. Therefore, they can be used as synthetic biomimetic receptors in sensing applications. The sensing performance of MIPs can be enhanced by micro- and nano-structuring because this increases the available recognition surface, greatly improving the site accessibility of imprinted materials [40]. The development of micro-nano-MIP-based chemical sensors normally requires the definition of well-defined MIP morphologies onto solid planar supports (e.g., transducers). For this purpose, a variety of methods for MIP film micro/nano patterning have been proved. Researchers have fabricated MIP features with microscale lateral resolution by means of UV [41], evanescent wave [42] and microstereo [43] lithographic techniques. However, to achieve sub-micron lateral resolution, only a few methods have been reported: Nanoimprint lithography [44] and direct writing by EBL [45].

**Figure 8:**
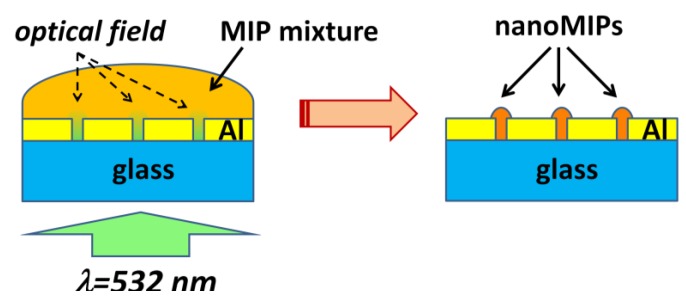
Schematics of the synthesis of nanoMIPs in Al nanoholes by local photopolymerization. (**Left**) A prepolymerization MIP mixture, containing a photoinitiator for photopolymerization at 532 nm wavelength, is deposited on an array of nanoholes and green light is applied from the glass substrate side. This leads to highly localized optical fields in the nanoholes; (**Right**) NanoMIPs after removing the non-polymerized mixture.

**Figure 9:**
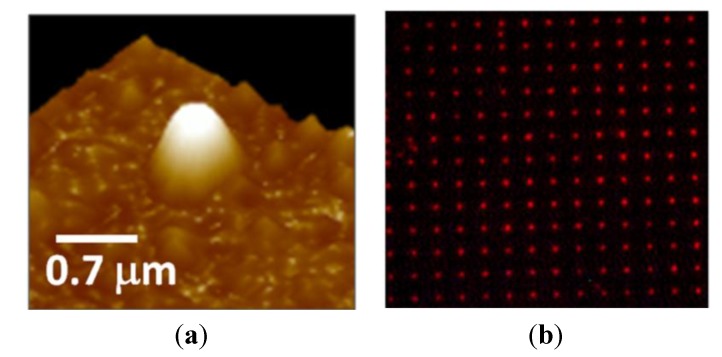
(**a**) AFM image of a single nanoMIP synthesized in an Al nanohole by local photopolymerization; (**b**) Fluoresce image of a 5-µm-period array of nanoMIPs containing fluorescent R123.

We have demonstrated a new technique for fabricating MIP patterns with sub-micrometer lateral resolution: Photoinduced local polymerization within Al nanoholes [46]. The fabrication procedure is schematically depicted in Figure 8. First, 2D arrays of Al nanoholes were fabricated on glass substrates, as described in Section 2. The period of the arrays was large enough to avoid optical interaction (interference effects) among the holes so they could be considered as isolated metal-clad circular waveguides. Then, the holes where wetted with a droplet of a MIP prepolymerization mixture that included a photoinitiator for photopolymerization at 532 nm wavelength and rhodamine 123 (R123) as a model template molecule. Next, a laser beam at 532 nm wavelength was incident normally to the Al nanoholes from the glass substrate side inducing local photopolymerization within the holes. Finally, the non-polymerized MIP mixture and the print molecule were removed by washing. The size of the resulting MIP nanomaterials at the nanoholes could be controlled by the dose of the green radiation, that is, by the exposure time and the incident light power. Figure 9 shows an atomic force microscope (AFM) image of a single nanoMIP synthesized in an Al nanohole (Figure 9a) and fluorescence emission from a 5-µm-period array of R123-containing nanoMIPs (Figure 9b). Fluorescence lifetime imaging microscopy (FLIM) with single photon timing measurements proved the capability of the fabricated MIP nanostructures to recognize selectively the target analyte R123 over rhodamine 6G and fluorescein analogs.

## 6. Conclusions

The use of Al as a low-cost plasmonic material is receiving increasingly attention due to the better understanding of its peculiarities related to the formation of aluminum oxide. Concerning biosensing, degradation of thin Al films because of corrosion and pitting has, however, placed this metal at a clear disadvantage as compared to the typically employed plasmonic metals Au and Ag. In order to circumvent this, we have developed a surface passivation treatment for nanopatterned Al films that allows Al NHA plasmonic transducers to be used for both, bioreceptor immobilization and target recognition in aqueous solutions. We have employed this technology to fabricate and demonstrate Al NHA biosensors on both, glass and PC CD supports. The latter achievement is particularly promising for developing CD-based label-free biosensing platforms for high-density microarray analysis. We have also shown the possibility of implementing Al NHAs on a flexible support, such as conventional Scotch tape using a simple stick-and-peel transfer procedure, which paves the way towards wearable devices based on Al devices combining plasmonic and electronic functionalities. Finally, the capability of isolated Al nanoholes to act as metal-clad circular optical waveguides has been employed to synthesize sub-micron sized MIP by means of local photopolymerization with visible radiation in the nanoholes. We consider that these achievements will contribute to make Al the material of choice for fabricating photonic transducers for biosensing applications, which would drastically enhance the possibility of commercialization of this type of optical biosensors.
